# Impact of Early and Regular Mobilization on Vital Signs and Oxygen Saturation in Patients Undergoing Open-Heart Surgery

**DOI:** 10.21470/1678-9741-2019-0481

**Published:** 2021

**Authors:** Sema Köse, Gülçin Avşar

**Affiliations:** 1 Nursing Services Directorate, Coordinator Nurse, Atatürk University Hospital, Erzurum, Turkey.; 2 Department of Nursing Fundamentals, Atatürk University Faculty of Nursing, Erzurum, Turkey.

**Keywords:** Cardiac Surgical Procedures, Heart Rate, Systole, Vial Signs, Reference Values, Intensive Care Units

## Abstract

**Introduction:**

This quasi-experimental study aimed to evaluate the impact of early and regular mobilization on vital signs and oxygen saturation in open-heart surgery patients.

**Methods:**

The study universe comprised patients undergoing open-heart surgery in the cardiovascular intensive care unit of a heart center. The study sample consisted of patients who underwent open-heart surgery from November 2016 to April 2017, met the inclusion criteria, and voluntarily agreed to participate in the study. The study included 75 patients. Of these, 67 completed the mobilization program in two days, starting on the first postoperative day. Each patient was mobilized three times: twice on the first postoperative day and once on the second postoperative day. Vital signs and oxygen saturation for each patient were measured 10 minutes before and 20 minutes after each mobilization.

**Results:**

The difference between pulse and systolic blood pressure values measured before and after the first mobilization was statistically significant (*P*<0.05). In addition, the difference between the mean systolic blood pressure values before the first mobilization and after the third mobilization (123.43±14.09 mmHg and 117.94±14.05 mmHg, respectively) was statistically significant (*P*<0.05). The other parameters measured in relation to the mobilizations were in the normal range.

**Conclusion:**

Early and frequent mobilization did not cause vital signs and oxygen saturation to deviate from normal limits in open-heart surgery patients.

**Table:** 

Abbreviations, acronyms & symbols	
**BMI**	**= Body mass index**
**ECG**	**= Electrocardiogram**
**ICU**	**= Intensive care unit**
**SpO_2_**	**= Oxygen saturation**
**SPSS**	**= Statistical Package for the Social Sciences**

## INTRODUCTION

Despite considerable changes in the concept and scope of nursing, the education level of nurses, the nature of the profession and the job description, roles, and functions of nurses, the only thing that has not changed is the provision of nursing care^[^^1^^]^. Intensive care units (ICUs) are one of the inpatient units, where nursing care is provided most intensively. Especially surgical ICUs represent the quality and adequacy of post-operative care, reflecting the success of the operation and playing a significant role in the prevention of potential complications^[^^2^^]^. The quality of care is life-saving after open-heart surgery, which is a significantly complex surgical intervention^[^^3^^]^.

Open-heart surgery is different from other surgical interventions, as blood is taken out of the body and delivered to the extracorporeal circulation while the lungs collapse and the heart temporarily stops^[^^4^^]^. Even elderly individuals with chronic diseases undergo open-heart surgery; however, these interventions are associated with high rates of complications, both in intraoperative and postoperative periods. The most important goal in postoperative care is to ensure a normal patient recovery as much as possible^[^^5^^]^. To achieve this goal, the improvement of cardiovascular functions is critical after open-heart surgery, along with maintaining tissue perfusion and vital signs, as well as maintaining respiratory functions through chest drainage and ventilation. Besides that, pain relief, maintenance of neurological functions, provision of psychological support, mobilization and prevention of potential postoperative complications are significantly important for the patient to achieve a successful recovery period^[^^6^^-^^9^^]^. Postoperative respiratory complications are among the most important factors that increase mortality and morbidity^[^^10^^]^. Early mobilization is the most effective action to rehabilitate the respiratory system^[^^4^^]^ to prevent the harmful effects of immobilization and bed rest in the postoperative period. Postoperative patient mobilization increases functional capacity, decreases oxygen consumption, reduces systolic and diastolic pressures in the left ventricle, and contributes to the regulation of the lipid and carbohydrate metabolism. In addition, mobilization is quite effective in increasing lung volumes, facilitating mucociliary clearance, maintaining the normal fluid distribution in the body, enhancing the level of consciousness, and assisting the patient to feel better psychologically^[^^6^^,^^11^^]^.

Although the positive effects of mobilization in open-heart surgery patients are known, early mobilization protocols for application in ICUs in the routine clinical practice have not been established^[^^11^^,^^12^^]^. Moreover, studies investigating the effects of early and regular mobilization strategies after different surgical interventions are available in the literature; however, no study has yet examined the impact of early, regular mobilization on vital signs and oxygen saturation after cardiac surgery. From this viewpoint, this study, evaluating the impact of early and regular mobilization on vital signs and oxygen saturation in open-heart surgery patients, is important, since it will contribute to knowledge in the field of nursing and will pave the way for further studies.

## METHODS

### Study Design

This study was conducted as a quasi-experimental study.

### Study Location and Date

The study was conducted in the Cardiovascular Surgery ICU of Ataturk University Health Research and Application Center from September 2016 to November 2018. There were 8 beds in the ICU; the number of staff nurses was six and, on average, two open-heart operation patients are admitted daily to the unit in the period of data collection for the study.

### Study Population and Sample

The study population consisted of patients who underwent open-heart surgery in the Cardiovascular Surgery Intensive Care Unit (ICU) of Ataturk University Health Research and Application Center. The study sample included 75 patients who underwent open-heart surgery in the period from November 2016 to April 2017 and who met the following inclusion criteria:

- No orthopedic and neurological problems.
- No inotropic support.
- No pulmonary embolism or deep vein thrombosis.
- Ability to speak Turkish.
- A Glasgow Coma Scale score of 13 or more.
- Age 18 years or older.

The exclusion criteria were:

- Inability to tolerate the mobilization program.
- Need for inotropic support during the mobilization program.
- Discharged from the ICU earlier than expected.
- Delirium during the mobilization program.
- Disagreement with participating in the mobilization program.

### Data Collection Tools

- Sociodemographic Information Form: a form developed by the researcher based on information in the literature^[^^6^^,^^13^^,^^14^^]^, documenting patient demographic information, such as age, gender, marital status, etc.
- Data Collection Chart: the vital signs and oxygen saturation values of the study patients were recorded regularly in this chart.
- Patient monitoring system devices and equipment: these tools (blood pressure cuff, saturation probe, heat probe, ECG cable, electrodes) regularly measured vital signs and oxygen saturation of patients in the ICU. They are calibrated every six months.

### Data Collection

The mobilization program started on the first postoperative day of the patients included in the study sample and lasted for two days. Each patient underwent a total of three mobilization sessions, performed in the following time intervals:

✓ From 10 am to 12 pm on the first postoperative day (it was recommended that patients be mobilized in the first 24 hours after operation within the scope of the "fast track" protocol, provided that their vital signs were stable^[^^15^^]^.
✓ From 12 pm to 2 pm on the first postoperative day.
✓ From 10 am to 12 pm on the second postoperative day.

**Fig. 1:**
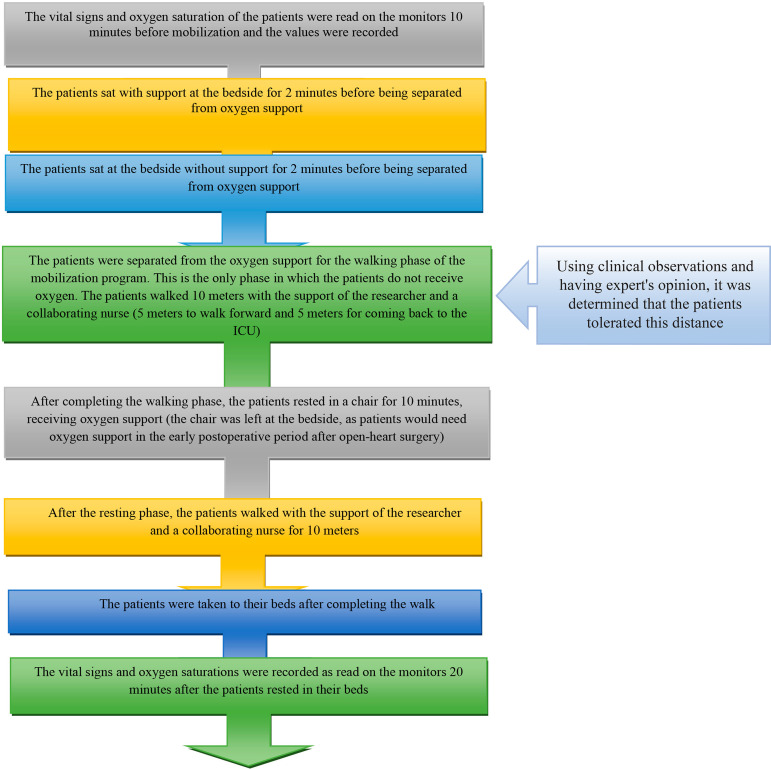
Study protocol.

The vital signs and oxygen saturation levels of the patients read on the monitors 10 minutes before and 20 minutes after each mobilization session were recorded on the data collection chart. The study protocol was carried out in the order described in Figure 1).

Mobilization is a painful process, demanding patient efforts after open-heart surgery^[^^15^^]^ and these patients need abundant quantities of oxygen in the early postoperative period. To obtain valid measures, patients were allowed to rest long enough, receiving adequate amounts of oxygen. Therefore, the adequate duration of bed rest was determined at 20 minutes.

**Fig. 2:**
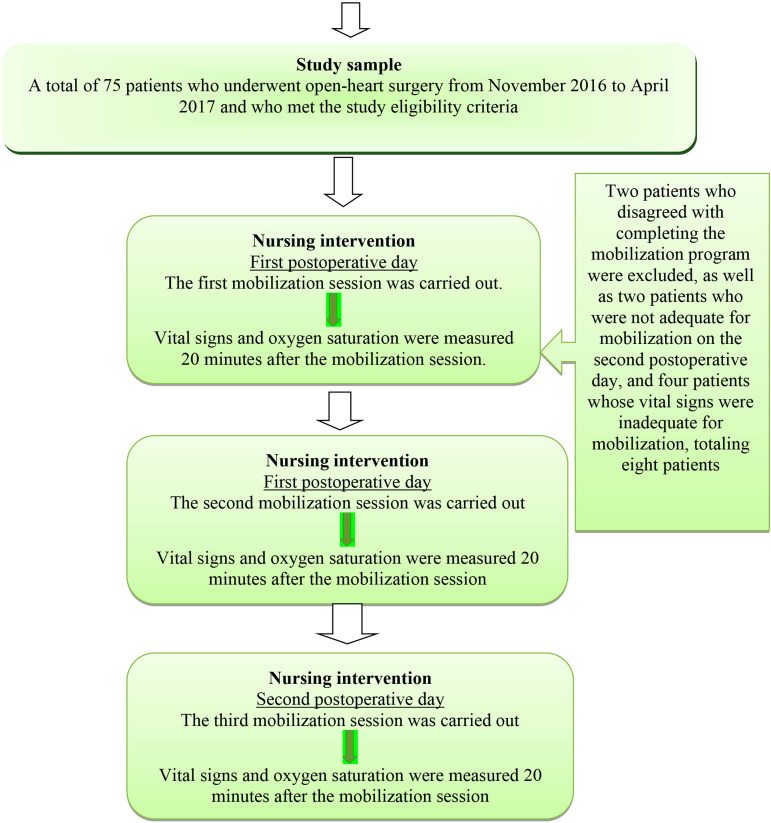
Study plan.

The order previously described was carried out three times in total. The first session was performed before noon and the second session was performed in the afternoon of the first postoperative day. Finally, the third session was carried out before noon on the second postoperative day (Figure 2).

### Data Assessment

The data were analyzed with SPSS package software version 17 for Windows. The numbers, percentages, minimum and maximum values, and mean and standard deviations were used to summarize the study data besides the statistical analyses.

#### Statistical Tests Used in the Analysis

- numbers, percentages, minimum and maximum values, and mean and standard deviations.
- Comparison of the values of pulse, diastolic blood pressure, systolic blood pressure, respiratory rate, body temperature, and SpO_2_ measured before and after the mobilization sessions: dependent sample t-test, mean and standard deviations, Wilcoxon test.

### Ethical Considerations

To conduct the study, approval of the Ethics Committee of Ataturk University Health Sciences Institute and legal permission from Ataturk University's Research Hospital were obtained. The ethical principle of "Respect for Autonomy" was fulfilled by informing the eligible patients about the purpose of the study and obtaining informed consent from patients who voluntarily agreed to participate in the study. The ethical principles of "confidentiality and preservation of confidentiality" were fulfilled by declaring that the information obtained would be kept confidential and used only for scientific purposes. The ethical principles of "beneficence and non-maleficence" were generally applied in the study.

### RESULTS

The study sample consisted of 75 patients who underwent open-heart surgery in the period from November 2016 to April 2017 and who met the study eligibility criteria. However, two patients who disagreed with completing the mobilization program were excluded, as well as two patients who were not adequate for mobilization on the second postoperative day, and four patients whose vital signs were inadequate for mobilization, totaling eight patients. Therefore, a total of 67 patients completed the study.

It was found that 47.8% of the patients were 61 years old or older; 73.1% were male and 44.8% had completed primary school. Of the study patients, 89.6% were married, 34.3% had normal body mass index (BMI) values, and 52.2% worked actively (Table 1).

**Table 1:** Demographic characteristics of patients (n=67).

Characteristics		N	%
Age	≤50	12	17.9
	51-60	23	34.3
	≥61	32	47.8
Gender	Female	18	26.9
	Male	49	73.1
Educational level	Illiterate	12	17.9
	Primary school	30	44.8
	Secondary school	7	10.4
	High school	14	20.9
	University	4	6.0
Marital status	Married	60	89.6
	Single	7	10.4
BMI	Underweight	2	2.9
	Normal	23	34.3
	Overweight	21	31.3
	Class 1 obesity	16	23.8
	Class 2 obesity	5	7.4
Working actively	Yes	35	52.2
	No	32	47.8
Other diseases	Yes	35	52.2
	No	32	47.8
Disease comorbidities	Diabetes	16	23.9
	COPD	2	3.0
	Hypertension	8	11.9
	Prostatic hypertrophy	6	9.0
	Other	15	22.5
Operation type	CABG	47	70.1
	Valve replacement	17	25.4
	Other	3	4.5

CABG=coronary artery bypass grafting; COPD=chronic obstructive pulmonary disease

Table 2 presents the comparison of the vital sign and oxygen saturation values measured before and after the first mobilization. The mean pulse values were 91.66±12.50 per minute before the first mobilization and 88.22±10.74 per minute after the mobilization. The difference between these values was statistically significant (*P*<0.05). After the mobilization session, the pulse value was decreased. The mean systolic blood pressure was 123.43±14.09 mmHg before the first mobilization and 117.67±13.38 mmHg after the first mobilization. The difference between these values was statistically significant (*P*<0.05). It was found that the systolic blood pressure decreased after the mobilization (Table 2).

**Table 2:** Comparison of vital signs and oxygen saturation in patients before and after first mobilization (n=67).

	Before first mobilization	After first mobilization	
Vital signs and SpO_2_	Mean±SD	Mean±SD	Significance
**Pulse rate**	91.66±12.50	88.22±10.74	t=4.090
			*P*=0.000
**Diastolic blood pressure**	59.19±8.30	58.43±9.39	t=0.849
			*P*=0.399
**Systolic blood pressure**	123.43±14.09	117.67±13.38	t=4.220
			*P*=0.000
**Respiration rate**	25.03±6.69	24.37±6.19	Z=-1.076
			*P*=0.282
**Body temperature**	36.86±0.49	36.90±0.48	t=-0.881
			*P*=0.382
**SpO_2_**	94.63±3.66	94.87±3.21	t=-0.798
			*P*=0.428

Table 3 presents the comparison of vital signs and oxygen saturation levels of the study patients before and after the second mobilization session. The mean pulse values of patients before and after the second mobilization session were 87.42±10.96 and 85.82±11.40 per minute, respectively. The difference between these values was statistically significant (*P*<0.05). The mean pulse after the session was lower compared to the value measured before the mobilization session. The mean diastolic blood pressure values of the patients before and after the second mobilization session were 60.28±10.99 mmHg and 60.69±10.45 mmHg, respectively. The difference between these values was not statistically significant (*P*>0.05) (Table 3). In addition, the differences between respiratory rate, systolic blood pressure, body temperature, and SpO_2_ values before and after the second mobilization were not statistically significant (*P*>0.05) (Table 3).

**Table 3:** Comparison of vital signs and oxygen saturation levels obtained before and after the second mobilization session (n=67).

	Before second mobilization	After second mobilization	
Vital signs and SpO_2_	Mean±SD	Mean±SD	Significance
**Pulse rate**	87.42±10.96	85.82±11.40	T=2.274
			*P*=0.026
**Diastolic blood pressure**	60.28±10.99	60.69±10.45	Z=-0.328
			*P*=-0.764
**Systolic blood pressure**	118.94±17.08	117.57±16.56	T=0.685
			*P*=0.496
**Respiratory rate**	25.64±6.35	24.48±7.07	T=1.618
			*P*=0.110
**Body temperature**	36.78±0.55	36.84±0.53	T=-1.498
			*P*=0.139
**SpO_2_**	94.63±5.69	95.31±3.34	Z=0.743
			*P*=0.445

Table 4 compares vital signs and SpO_2_ values before and after the third mobilization. The mean diastolic blood pressure values of the patients before and after the third mobilization session were 61.91±9.78 mmHg and 59.88±8.89 mmHg, respectively. The difference between these blood pressure values was not statistically significant (*P*<0.05), with lower blood pressure values after the session compared to the previous values (Table 4). The differences between respiratory rate, systolic blood pressure, body temperature and SpO_2_ values before and after the third mobilization were not statistically significant (*P*>0.05) (Table 4).

**Table 4:** Comparison of vital signs and oxygen saturation levels obtained before and after the third mobilization session (n=67).

	Before third mobilization	After third mobilization	
Vital signs and SpO_2_	Mean±SD	Mean±SD	Significance
**Pulse rate**	91.46±12.94	91.21±11.89	T=0.341
			*P*=0.734
**Diastolic blood pressure**	61.91±9.78	59.88±8.89	T=2.029
			*P*=0.047
**Systolic blood pressure**	121.55±16.69	117.94±14.05	T=2.337
			*P*=0.023
**Respiratory rate**	25.49±5.76	24.94±5.63	T=0.651
			*P*=0.517
**Body temperature**	36.81±0.54	36.86±0.52	T=-0.953
			*P*=0.344
**SpO_2_**	94.96±3.79	94.48±3.56	Z=-1.856
			*P*=0.064

Table 5 shows the comparison of vital signs and oxygen saturation levels of the patients before the first and after the third mobilization sessions. The mean systolic blood pressure values of the patients were 123.43±14.09 mmHg before the first mobilization and 117.94±14.05 mmHg after the third mobilization, with a statistically significant difference (*P*<0.05). The systolic blood pressure after the third mobilization session was lower compared to the value measured before the first monitoring session. No statistically significant differences were found in pulse and respiratory rates, and in the values of diastolic blood pressure, body temperature and SpO_2_ before the first and after the third mobilization sessions.

**Table 5:** Comparison of vital signs and oxygen saturation of patients before the first mobilization and after the third mobilization (n=67).

	Before first mobilization	After third mobilization	
Vital signs and SpO_2_	Mean±SD	Mean±SD	Significance
**Pulse rate**	91.66±12.50	91.21±11.89	t=0.293
			*P*=0.770
**Diastolic blood pressure**	59.19±8.30	59.88±8.89	t=-0.628
			*P*=0.532
**Systolic blood pressure**	123.43±14.09	117.94±14.05	t=2.515
			***P*=0.014**
**Respiration rate**	25.03±6.69	24.94±5.63	t=0.102
			*P*=0.919
**Body temperature**	36.86±0.49	36.86±0.52	t=0.020
			*P*=0.984
**SpO_2_**	94.63±3.66	94.48±3.56	Z=-1.318
			*P*=0.187

## DISCUSSION

In this study, patients were mobilized three times in total: twice in the first 24 hours after the operation and once in the next 24 hours. The sessions took place between 10 am and 12 pm and 12 pm and 2 pm. The impact of mobilization on vital signs was examined in both sessions. The comparison of vital sign values and oxygen saturation levels in patients before and after the first mobilization did not reveal differences in diastolic blood pressure, respiratory rate, body temperature, and oxygen saturation values. Sala et al.^[^^14^^]^ followed up heart rate and oxygen saturation during the early rehabilitation period in patients undergoing heart surgery. The authors reported that early mobilization was safe, without leading to negative outcomes for the patients. Bourdin et al.^[^^16^^]^ compared the applicability of early mobilization in ICU patients. They compared the physiological parameters obtained during the sitting and standing positions and before and after walking sessions. The study did not report any significant differences between heart rate, respiratory rate, mean arterial pressure, and oxygen saturation values obtained. Our study found similar results and found no negative outcomes from early mobilization. These results demonstrated that the gradual mobilization of the patients maintained normal vital signs after open-heart surgery, suggesting that the mobilization was applicable. However, statistically significant reductions were observed in the pulse rate and systolic blood pressure. In the literature, reductions in systolic blood pressure and pulse rate are evaluated as orthostatic hypotension, along with the cardiovascular stress symptoms, suggesting that the cardiovascular system cannot tolerate mobilization after a major cardiac operation^[^^16^^,^^17^^]^. Based on literature information, the reductions observed in pulse rate and systolic blood pressure in our study were considered due to the cardiovascular instability present in the first mobilization session.

The comparison of vital signs and oxygen saturation levels of the patients before and after the second mobilization session did not show significant differences in vital signs and oxygen saturation. These values were within the normal reference limits; however, the pulse rate was statistically significantly reduced after the second mobilization session. The study by Genç^[^^17^^]^ examined the effects of mobilization on the cardiopulmonary system of ICU patients. The study found that there were no significant changes in systolic and diastolic blood pressure values occurred when the patients stood up and walked compared to the values obtained from patients in the supine position. Şenduran et al.^[^^18^^]^ gradually mobilized liver transplant patients after surgery and reported increases in the heart rate during all stages of mobilization. Furthermore, the authors reported that the observed elevation in heart rate was normalized within 5 minutes. In parallel with the literature studies, our study found no changes in vital signs, except a reduction in pulse rate when patients rested in bed after the mobilization session. It is reported in the literature that, during the mobilization session, reduced heart rate and symptoms of cardiovascular stress indicate non-tolerance to mobilization by the cardiovascular system^[^^16^^,^^17^^]^. This information suggests that the reduction in the pulse rate should be anticipated during the first postoperative day. In our study, the pulse rate decreased after the first and second mobilization sessions on the first postoperative day; however, no reductions in pulse rate were observed after the third mobilization session on the second postoperative day.

There were no significant changes observed in pulse rate, systolic blood pressure, respiratory rate, body temperature, and oxygen saturation levels when the vital signs and oxygen saturation levels of the patients obtained before and after the third mobilization session were compared. Contrary to our findings, another study, investigating the impact of early mobilization on vital sign stability, reported higher pulse and respiratory rates after mobilization compared to values measured before the mobilization session^[^^19^^]^. We consider that these findings of our study demonstrated that the mobilization session carried out on the second postoperative day contributed to the maintenance of vital signs within the normal reference range.

The comparison of the vital signs and oxygen saturation levels of the patients before the first and after the third mobilization sessions did not reveal statistically significant differences in pulse rate, diastolic blood pressure values, respiratory rates, body temperature, and oxygen saturation. The study by Yolcu et al.^[^^13^^]^ reported that post-mobilization values of blood pressure, pulse rate, and respiratory rate in the postoperative period were significantly higher compared to pre-mobilization values. In this study, no significant differences were detected in vital signs after the mobilization sessions, with all values within the normal reference limits. These results suggested that the early and regular mobilization program contributed to maintaining the stability of patients after extubation performed in the presence of favorable vital sign values. In addition, our results suggested that the early and regular mobilization program was effective in all parameters, including mainly those belonging to the respiratory and cardiovascular systems. However, in contrast to the traditional ICU practices, early rehabilitation is currently carried out when patients are minimally sedated or, if possible, no sedation is applied^[^^4^^,^^20^^]^.

Yolcu et al.^[^^13^^]^ reported that heart surgery patients experienced more problems during mobilization and needed more support in the postoperative period compared to orthopedic and general surgery patients. The results of that study suggested that mobilization helped to maintain vital signs within normal ranges and contributed to cardiac stability, demonstrating the applicability of the method. Furthermore, the study results are considered important, as they provided guarantees against the safety concerns associated with the applicability of mobilization, especially in this patient population. Moreover, the absence of alterations in vital signs and oxygen saturation levels after the mobilization sessions, despite the high frequency of patients aged 61 or older in the study, provides significant evidence that mobilization can be performed in this patient population. This information contributes considerably to the information in the literature, because it is reported that patients 65 years or older are at a greater risk of postoperative complications compared to younger patients^[^^21^^]^, are more prone to develop changes in pulmonary functions^[^^22^^]^, and some complications are observed at significantly higher rates in patients aged 60 years and older^[^^10^^]^ after cardiac surgery.

In the study, a statistically significant decrease was observed in the systolic blood pressure values of the patients when compared to the values obtained before the first and after the third mobilization session. However, these systolic blood pressure values were within normal ranges before and after the mobilization sessions. Considering that hypotension or hypertension is commonly reported in the literature after heart surgery^[^^5^^,^^23^^,^^24^^]^ and that the systolic blood pressure values in our study remained in the normal range, despite the observed reductions, we suggest that the early and regular mobilization strategy has positive effects. These results demonstrate that an early and regular mobilization program is applicable to patients in this population because it did not lead the vital signs and oxygen saturation values to deviate from normal reference values.

## CONCLUSION AND RECOMMENDATIONS

It was concluded that early and regular mobilization in open-heart surgery patients did not cause vital signs and oxygen saturation levels to deviate from normal values within the reference range and that it is usable in this patient population.

Based on these results, it may be recommended that:

- Early and regular mobilization should be included in the care protocols for patients undergoing open-heart surgery.
- Training about the importance of early and regular mobilization in open-heart surgery patients should be provided to healthcare professionals.
- Further studies should be conducted to investigate the effects and potential complications of early and regular mobilization programs in ICU patients, who underwent open-heart surgery.
- Further studies should investigate the effects of different mobilization programs.

**Table:** 

Authors' roles & responsibilities	
SK	Substantial contributions to the conception or design of the work; or the acquisition, analysis, or interpretation of data for the work; drafting the work or revising it critically for important intellectual content; agreement to be accountable for all aspects of the work in ensuring that questions related to the accuracy or integrity of any part of the work are appropriately investigated and resolved; final approval of the version to be published
GA	Substantial contributions to the conception or design of the work; or the acquisition, analysis, or interpretation of data for the work; drafting the work or revising it critically for important intellectual content; agreement to be accountable for all aspects of the work in ensuring that questions related to the accuracy or integrity of any part of the work are appropriately investigated and resolved; final approval of the version to be published

## References

[1] Taşocak G, Aştı TA, Karadağ A (2014). Nursing Fundamentals Nursing Science and Art.

[2] Yıldırım N, Atalay M (2002). Evaluation of the quality of life of patients with coronary artery bypass surgery. Res J Nurs.

[3] Pour HA, Korkmaz FD (2010). Nursing care after open heart surgery. J Ege Univ Sch Nurs.

[4] Morris PE, Herridge MS (2007). Early intensive care unit mobility: future directions. Crit Care Clin.

[5] Parkosewich JA, Smeltzer SC, Cheever KH, Hinkle JL, Bare BG (2010). Brunner and Suddarth's Textbook of Medical-Surgical Nursing.

[6] Badır A, Korkmaz FD, Karadakovan A, Aslan FE (2014). Care in Internal and Surgical Diseases.

[7] Craven RF, Hirnl C, Jensen S, Uysal N, Çakırcalı E (2015). Principles of Nursing Human Health and Functions.

[8] Black JM, Hawks JH (2009). Medical surgical nursing: clinical management for positive outcomes.

[9] Cook M, Idzior L, Bena JF, Albert NM (2017). Nurse and patient factors that influence nursing time in chest tube management early after open heart surgery: A descriptive, correlational study. Intensive Crit Care Nurs.

[10] Sargın A, Aşkar FZ, Kocabaş SN (2013). Preoperative, intraoperative and postoperative predictors of postoperative respiratory system complications in patients undergoing open heart surgery. GKD Anest Yoğ Bak Dern Derg.

[11] Stiller K, Phillips A, Lambert P (2004). The safety mobilisation and its effect on haemodynamic and respiratory status of intensive care patients. Physiother Theory Prac.

[12] Bailey P, Thomsen GE, Spuhler VJ, Blair R, Jewkes J, Bezdjian L (2007). Early activity is feasible and safe in respiratory failure patients. Crit Care Med.

[13] Yolcu S, Akın S, Durna Z (2016). The evaluation of mobility levels of postoperative patients and associated factors. HEAD.

[14] Sala V, Petrucci L, Monteleone S, Dall'Angelo A, Miracca S, Conte T (2016). Oxygen saturation and heart rate monitoring during a single session of early rehabilitation after cardiac surgery. Eur J Phys Rehabil Med.

[15] Vermişli S, Çam K (2015). The efficacy of early mobilization after urologic radical surgery. Bul Urooncol.

[16] Bourdin G, Barbier J, Burle JF, Durante G, Passant S, Vincent B (2010). The feasibility of early physical activity in intensive care unit patients: a prospective observational one-center study. Respir Care.

[17] Genç A (2007). Effects of Mobilization Programs Applied in Intensive Care Patients on the Cardiopulmonary System.

[18] Senduran M, Yurdalan SU, Karadibak D, Gunerli A (2010). Haemodynamic effects of physiotherapy programme in intensive care unit after liver transplantation. Disabil Rehabil.

[19] Özçelik Z, Uçar N, Yılmaz D, Koç N, Akıncı SB (2017). Administration of early mobilization in intensive care unit patients and effects of early mobilization on patient hemodynamics. J Turk Soc Intens Care.

[20] Latronico N, Rasulo FA (2010). Presentation and management of ICU myopathy and neuropathy. Curr Opin Crit Care.

[21] Higgins TL, Yared JP, Estafanous FG, Barash PG, Reves JG (2001). Cardiac Anesthesia.

[22] Walston JD, Pierre Michel J, Beattie BL, Martin FC, Walston JD (2018). Oxford Text¬book of Geriatric Medicine.

[23] Dressler DK, Smeltzer SC, Cheever KH, Hinkle JL, Bare BG (2010). Brunner and Suddarth's Textbook of Medical- Surgical Nursing.

[24] Vincent JL, Norrenberg M (2009). Intensive care unit-acquired weakness: framing the topic. Crit Care Med.

